# Presenting the Special Issue “Aquaporins: Dynamic Role and Regulation”

**DOI:** 10.3390/ijms26094384

**Published:** 2025-05-05

**Authors:** Marianna Ranieri, Grazia Tamma

**Affiliations:** Department of Biosciences, Biotechnologies and Environment, University of Bari, 70125 Bari, Italy; grazia.tamma@uniba.it

## 1. Introduction

Aquaporins (AQPs) were discovered in human erythrocytes in 1987 by Peter Agree and collaborators [1]. AQPs belong to a family of small integral membrane proteins involved in the transport of water [2] and other small solutes including glycerol, urea, and hydrogen peroxide [3].

The importance of aquaporins is related to their main function [4,5,6,7,8,9,10,11,12,13]. Water is the major component of cells and tissues and its movement through plasma membranes is essential for life. Until the discovery of the first water channel, it was long assumed that water transport occurred by simple diffusion through the membrane lipid bilayer. To date, great strides have been made towards a better understanding of the dynamic roles of these proteins. Based on their transport specificity, aquaporins can be classified into four groups [14]: (1) classical or orthodox AQPs, (AQP0, 1, 2, 4, and 5), which are water-selective [15]; (2) aquaglyceroporins (AQP3, AQP7, AQP9, and AQP10), which mediate water and glycerol transport [16]; (3) peroxiporins (AQP0, AQP1, AQP3, AQP5, AQP8, AQP9, and AQP11) [17,18]; and (4) unorthodox super aquaporins or subcellular aquaporins (AQP11 and AQP12) [19,20]. AQP6 belongs to the AQP family in terms of its aminoacidic sequence; however, it functions as an anion-selective channel [21].

Hence, aquaporins are involved in a wide range of physiological processes in diverse tissues due to their functions and expression pattern. Therefore, their abnormal function and trafficking can cause several diseases [22,23].

The present Special Issue, entitled “Aquaporins: Dynamic Role and Regulation”, provides new insight into aquaporins’ functions and involvement in different regulatory pathways, enabling greater understanding of water channel regulation, which is crucial for designing novel therapeutic approaches.

All manuscripts submitted for consideration for the Special Issue were subjected to a rigorous review process. Nine papers, comprising three reviews and six articles, were accepted for publication and are included in this Special Issue.

## 2. Overview of Included Articles

In the first paper, Kemény and Ducza [24] focused on the functional interaction between AQP5 and TRPV4 in several tissues and organs including the lungs, salivary glands, uterus, adipose tissues, and lens. Specifically, TRPV4 stimulation has been correlated with AQP5 membrane trafficking, which controls the osmotic water permeability of several tissues. Investigating this interaction would provide the opportunity to design new therapeutic tools to tackle different conditions such as cataracts, asthma, ischemia/reperfusion-induced edema, and COVID-19 lung disease, or could be used in the prevention of premature births.

D’Agostino C. et al. [25]’s excellent review provides a comprehensive overview of the current knowledge on the upstream and downstream effectors of AQP5, offering a detailed understanding of physiological and pathophysiological processes that involve AQP5. Novel findings have revealed that AQP5, besides water, could mediate the transport of hydrogen peroxide and CO_2_, thereby implicating this water channel in numerous intracellular pathways in health and disease. Among these, it is worth mentioning the single nucleotide polymorphisms, mutations, and transcriptional control of the Aqp5 gene that affect the protein’s expression, trafficking, and/or function. Furthermore, post-translational modifications; intracellular signaling pathways, mediated by calcium and/or cAMP-PKA; selective protein/protein interactions; cytoskeleton binding; and other mechanisms play a role in regulating AQP5 trafficking and localization.

Exploring the mechanism regulating inflammation and human sepsis, Rump K et al. [26], showed that AQP5 expression in neutrophil granulocytes correlated with survival rates associated with sepsis in humans. Specifically, a possible mechanism has been proposed, revealing that the AQP5 A/C-1364 promoter polymorphism impacts AQP5 expression by altering AQP5 promoter methylation. At a molecular level, this study could open new avenues into finding new targeted drugs affecting AQP5 promoter methylation and thereby have a clinical impact on our understanding of mechanisms underlying the regulation of inflammatory damage.

Petrova R.S. et al. [27] investigated the functional actions of pilocarpine-induced decreases in zonular tension, resulting in increased hydrostatic pressure, due to the removal of AQP5 from fiber cell membranes in the lens. They showed that these mechanisms are associated with TRPV1 channel activation. Importantly, the pilocarpine-induced removal of AQP5 from cellular membranes is prevented by TRPV1 inhibition, or, in the absence of pilocarpine, might be elicited by TRPV4 inhibition, which results in TRPV1 stimulation. Taken together, these new findings reveal an intricate functional interaction between AQP5, TRPV1, and TRPV4, controlling hydrostatic pressure in the lens.

Exploring the non-genomic effects of corticosteroids, Mom R. et al. [28] reported a putative AQP corticosteroid binding site (ACBS) by performing in silico molecular dynamics simulations. Several findings proposed the presence of novel membrane “receptors” involved in the short-term action of corticosteroids. Specifically, an in silico simulation analysis proposed ACBS spanning from the extra-cellular arginine of the ar/R constriction up to the end of the extra-cellular domains of AQPs where the flexible loops (C-loop and A-loop) are located. Also, the authors suggested that the interaction between the ACBS and corticosteroids might impact AQPs’ proprieties by limiting their ability to transport across the plasma membrane.

Calcium signaling plays a key role in controlling intracellular vesicle trafficking. Scorza S. et al. [29] investigated in depth the involvement of lysosomal Ca^2+^ signals, using mouse renal collecting duct (CD) cells as an experimental model. By applying different methodological approaches, the authors demonstrated that lysosomal Ca^2+^ signaling events are mediated by TRPML1. Also, lysosomal calcium release, through calcineurin signaling, mediates actin depolymerization and AQP2-mediated water reabsorption. Together, these new findings shed more light on the complex calcium signaling that controls AQP2-dependent water transport and propose TRPML1 as a novel player in AQP2 trafficking.

Over the years, AQP2 expression and trafficking have been investigated by using different renal cell models. mpkCCDc14 cells have been used for a long time to study the mechanisms controlling AQP2 expression. However, the low endogenous AQP2 protein content in the absence of the hormone vasopressin and differential AQP2 expression within cells prompted Jang H.-J. et al. [30] to establish V2R-AQP2 knock-in mpkCCDc14 cells using CRISPR/Cas9 genome-engineering technology. The engineered V2R-AQP2 cells express both the constitutively expressed AQP2 (integrated) and vasopressin-responsive AQP2 endogenously expressed in mpkCCDc14 cells. The generation of this new cell model would be useful to further dissect the complex machinery controlling the expression, localization, and function of the vasopressin-dependent water channel AQP2.

Numerous reports have demonstrated that increased AQP3 expression is related to tumor progression and, thus, it is considered a potential target in cancer therapy. Mlinarić M. et al. [31] investigated the putative involvement of the peroxiporin Aquaporin 3 (AQP3), which transports hydrogen peroxide, water, and glycerol in tumorigenic and nontumorigenic cell lines. Interestingly, in the nontumorigenic cell line, MCF10A, neither AQP3 silencing nor EGF treatment affected the intracellular pathways. By contrast, AQP3 was found to play a role in PI3K/Akt activation and oxidative effects in tumorigenic cell lines, albeit in a cell-line-dependent manner.

The prevalence of infertility, a common health disease having heterogeneous etiologies, is growing globally. Nunes D.C. et al. [32] summarized the main findings related to the involvement of AQP-mediated function and permeability in men with metabolic syndrome and testosterone deficit. They highlight possible mechanisms explaining the interaction between sex hormones, AQPs, and metabolic syndrome, which may contribute to male infertility.

## 3. Conclusions

This Special Issue presents several studies that shed light on aquaporin expression and function. Covering a wide spectrum of fields, ranging from cell physiology, transport, diseases, and cancer, each manuscript provides a novel perspective, contributing to our understanding of AQPs’ involvement in complex transduction pathways in health and disease.
